# Data on the physicochemical characteristics and texture classification of soil in Bornean tropical heath forests affected by exotic *Acacia mangium*

**DOI:** 10.1016/j.dib.2023.109670

**Published:** 2023-10-11

**Authors:** Salwana Md. Jaafar, Rahayu Sukmaria Sukri

**Affiliations:** Institute for Biodiversity and Environmental Research, Universiti Brunei Darussalam, Bandar Seri Begawan BE 1410, Brunei

**Keywords:** Brunei, Edaphic properties, Exotic plants, invasive *Acacia*, Nutrients, Physical composition, Plantation, soil properties

## Abstract

This article describes distinctive soil properties within three Bornean tropical heath forest habitats associated with *Acacia* invasion in Brunei Darussalam. The data was systematically collected from eighteen 20 × 20 m plots set up within an *Acacia mangium* plantation, the adjacent intact heath forest, and *Acacia mangium* invaded heath forest. Within each plot, we collected eight soil cores from both topsoil and subsoil depths and measured soil pH, soil gravimetric water content, organic matter content, and concentrations of soil nutrients (total nitrogen, phosphorus, potassium, magnesium, and calcium, as well as exchangeable magnesium, calcium, and potassium and available phosphorus). Soil texture classification across all three habitats were also determined. This comprehensive dataset offers valuable insights into the ecological consequences of *Acacia* invasion into Bornean heath forests. Given the scarcity of studies focusing on diverse Bornean soil types and the impacts of invasive plants, our dataset can supplement future research efforts. Consequently, this dataset holds considerable value as a tool to offer insights to effectively address the challenge posed by exotic plant invasions on native tropical ecosystems.

Specifications Table

**Table:** 

Subject	Environmental Science, Ecology, Nature and Landscape Conservation
Specific subject area	Edaphic properties, plant invasion, soil science
Data format	Raw and filtered
Type of data	Table .csv files (dataset with labels)
Data collection	At each habitat type, six 20 × 20 m plots were set up and further sub-divided into four 10 × 10 m subplots. Within each subplot, one soil core was sampled using a soil auger both at the topsoil (0–15 cm) and subsoil (30–50 cm) depths. Fresh soils were analysed for pH and GWC. The remaining fresh soil samples were then air-dried, ground, and passed through a 2.0 mm sieve. Samples were then analysed for concentrations of total N, P, Mg, Ca, K, exchangeable Mg, Ca, K, and available P. Organic matter was determined using a muffle furnace and soil texture was determined using a modified pipette method.
Data source location	Soils were collected from three different heath habitat types in Brunei Darussalam, northwest Borneo: intact heath forest, locally referred to as ‘Kerangas’ (4°35′59.7″N, 114°30′58.3″E), an *Acacia mangium* plantation (4°35′44.9″N, 114°30′51.7″E), and *Acacia*-invaded heath forest adjacent to the plantation (4°35′34.5″N 114°31′308″E).
Data accessibility	Repository name: Zenodo Data identification number: 10.5281/zenodo.8389823 Direct URL to data: https://zenodo.org/record/8389823
Related research article	No related article has been published to date.

## 1 Value of the Data


 

• This dataset serves as a valuable reference for ecological studies on the invasion of alien plant species in Borneo. They offer a distinct perspective into the dynamics of degraded Bornean heath forest resulting from the invasion of *Acacia* species.
• Currently, there is a scarcity of publicly available datasets on edaphic properties in Brunei Darussalam and Borneo, and our datasets can serve as a valuable resource for comparison with other tropical forest types within Borneo.
• The dataset holds significance for ecologists, soil scientists, geologists and environmental scientists engaged in research within the tropical rain forest region, particularly in Borneo. The data can be incorporated into ecological models used in forest conservation and climate change studies, and utilized to guide restoration and rehabilitation projects. Additionally, our datasets can be used in meta-analyses to elucidate the impact of invasive species on soil properties on forested ecosystem worldwide, allowing wider utility of our dataset in diverse ecological and environmental research.

## 2 Objective

Among invasive plants, the genus *Acacia* is particularly invasive and is often regarded worldwide as a one of the worst plant invaders [1], [2], [3], [4]. Notably, nitrogen-fixing Acacias possess a distinct capability to modify soil nutrient dynamics, thus augmenting their invasive prowess [5], [6], [7], [8], [9]. In Brunei Darussalam, *Acacia mangium* that were initially introduced into timber plantations and as roadside plantings are increasingly spreading into disturbed forest habitats, in particular the heath forests [10,11]. They have been recognized for their ability to increase the deposition of calcium (Ca), magnesium (Mg), and ammonium (NH_4_^+^) ions in soil [12]. Additionally, they lead to alterations in soil profiles, lowered soil bulk density, decreased soil moisture, organic matter, and base cations, while increasing soil temperature and pH [13]. Moreover, they elevate gravimetric water content and the concentrations of soil phosphorus (P), potassium (K), and Ca [14]. This also results in increased production of litterfall and higher concentrations of nitrogen (N), K, and Ca in leaf litter, alongside reducing the use efficiencies in N and K [15].

## 3 Data Description

**Fig. 1:**
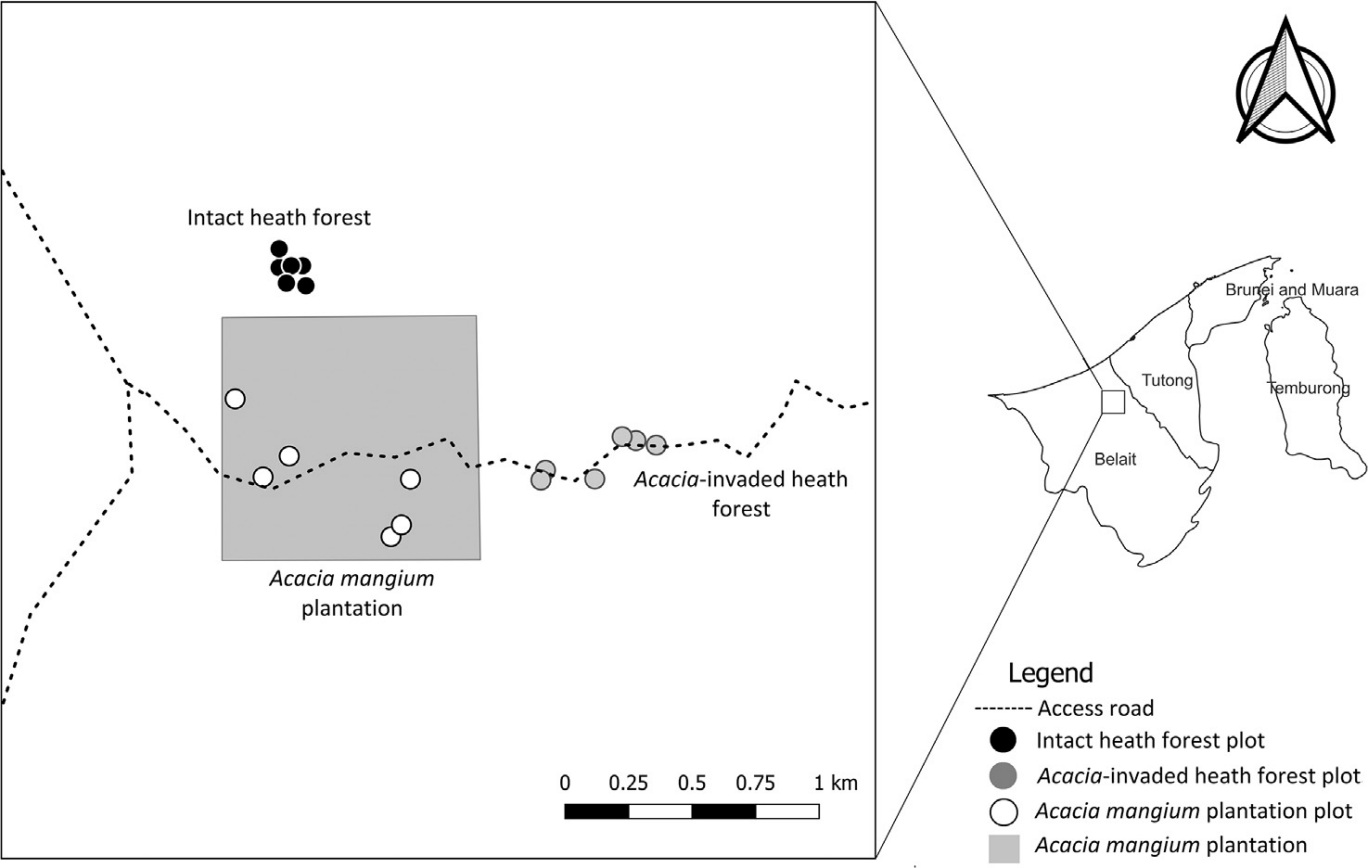
Locations of the study sites consisting of six plots in *Acacia mangium* plantation (white dots), six plots in intact heath forest (black dots) and six plots in *Acacia*-invaded heath forest (gray dots) within the compartment 8 of Andulau Forest Reserve in Sungai Liang, Brunei Darussalam.

This data article reports a new dataset of soil physical and chemical properties and texture classifications used to understand the impacts of *A. mangium* species invasion in Bornean lowland heath forest. The dataset was collected as part of a comprehensive research project investigating the impact of invasive *Acacia* species on different tropical forests in Brunei Darussalam [12], [13], [14], [15]. Fig. 1 presents three distinctive habitats within the Andulau Forest Reserve in Sungai Liang in Belait district, Brunei Darussalam, namely *A. mangium* plantation, the adjacent intact heath forest, and the *Acacia*-invaded heath forest.

The dataset is stored in a data repository storage in comma-separated values (.csv) table format, called GPS.csv, Soil physicochemical properties.csv and Soil texture classification.csv. The GPS.csv file contains the information of the plot's location with GPS coordinates (WGS 84 system) presented in decimal degree notation. Soil physicochemical properties.csv provide details on physical properties of soil including the gravimetric water content (GWC) and organic matter (OM) in percentage (%), and nutrients concentrations including total N, P, K, Mg, Ca, exchangeable K, Mg, Ca, available P in milligrams per gram (mg/g) and pH. Soil texture classification.csv file contains information of soil texture through the calculation of silt, clay and sand in percentage (%). Data was acquired via field sampling, measurement and chemical analysis and the formats given are raw and filtered data. In detail, 18 plots were set up, 144 soil samples were collected, and 13 soil parameters were analyzed (Table 1).

**Table 1:** Summary of the type and volume of data provided. This data article contains three .csv files.

	GPS	Soil physicochemical properties	Soil texture classification
Number of points	18	72	72
Number of samples	–	144	144
Number of samples per soil depth	–	72	72
Number of parameters	–	13	13
Sampling period	April 2015		

## 4 Experimental Design, Materials and Methods

Fieldwork was conducted in April 2015, within three weeks during the dry season from February to May 2015. A total of 144 soil samples were collected, involving 72 subplots at two different depths, from 18 plots located within three distinct habitat types. Within each of these habitat types, six plots measuring 20 × 20 m were set up, which were subsequently divided into four 10 × 10 m subplots. The plots in each habitat type were positioned at distances more than 50 m from one another, as illustrated in Fig. 1.

**Fig. 2:**
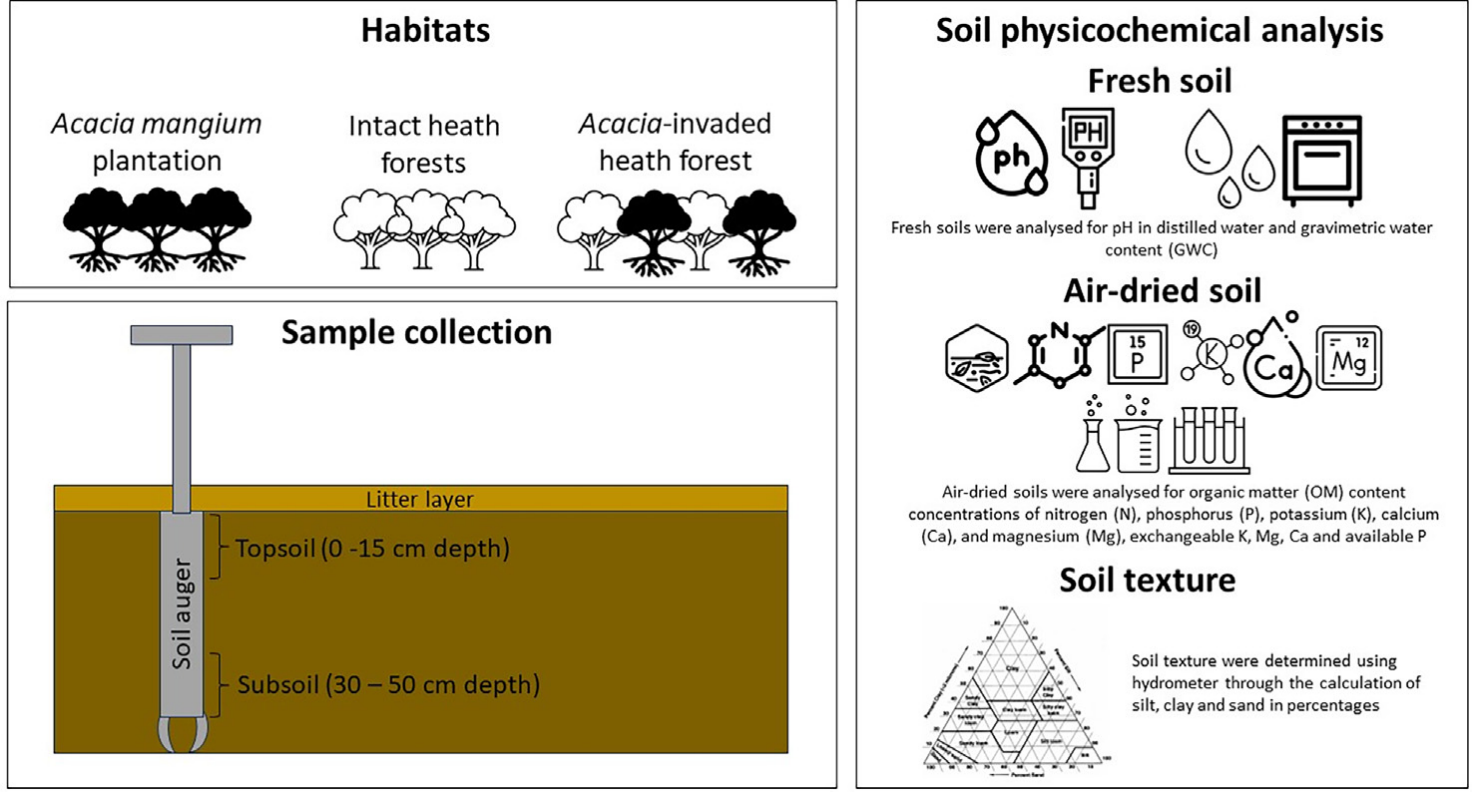
Graphical overview of the habitat variables, sample collection and parameters included in the dataset.

Within each of the subplots, one soil core was randomly obtained using a soil auger from both the topsoil layer (0–15 cm) and the subsoil layer (30–50 cm), as shown in Fig. 2. The freshly collected soil samples underwent analysis for pH in distilled water and gravimetric water content (GWC) following standard protocol by Allen et al. [16] and Jaafar et al. [17]. The pH of the soil (10 g fresh soil mixed with 20 mL distilled water) was determined using a bench-top pH meter (Hanna Instruments Ltd, based in Bedfordshire, UK). The GWC was determined by subjecting approximately 10 g of fresh soil to oven-drying at a consistent weight of 105 °C for 24 h [16,17]. The remaining fresh soil samples were air-dried, crushed, and sieved using a 2.0 mm sieve, and stored at room temperature for further analyses.

The determination of organic matter (OM) content (about 10 g) was carried out using a muffle furnace (Gallenkamp Size 2, Apeldoorn, Netherlands) set to 550 °C for a duration of two hours [16,17]. Soil texture was analyzed using a modified pipette method, following the procedures outlined by the Brunei Department of Agriculture [18].

The soil samples underwent comprehensive analysis for concentrations of various elements, including total N, P, Mg, Ca, K, exchangeable Mg, Ca, K, and available P. Concentrations of total N and P were determined using the Kjeldahl method, through the digestion of each soil sample in concentrated sulphuric acid for 2 h at a temperature of 360 °C, followed by analysis using a Flow Injector Analyser (FIAstar 5000, Hoganas, Sweden). For the determination of total Mg, Ca, and K concentrations, air-dried soil samples were acid-digested using a microwave digestor (Multiwave 3000 Anton Paar, Austria), following Allen et al. [16]. The extraction of exchangeable Mg, Ca, and K was performed using 1 N neutral ammonium acetate [19]. Measurements of total and exchangeable Mg, Ca, and K concentrations were conducted using a Flame Atomic Absorption Spectrophotometer (AAS; Thermo Scientific iCE 3300, Sydney, Australia). The assessment of soil available P concentrations involved the use of Bray's solution (0.03 N ammonium fluoride in 0.025 N HCl), combined with ascorbic acid and molybdate reagent [17,18]. The absorbance was read at a wavelength of 880 nm using a UV-spectrophotometer (UV-1800, Shimadzu, Kyoto, Japan).

## 5 Limitations

While our dataset provides valuable insights into the physicochemical characteristics of soil in the three different habitats studied, we acknowledge the possibility of spatial autocorrelation among the plots within each of the three habitats. To reduce spatial autocorrelation, plot distances within a habitat were kept to a minimum distance of 50 m. Further, plots within a habitat were set up at random locations to function as independent sampling units. We note that spatial autocorrelation can introduce potential dependencies in the data and recommend this to be considered in the use and statistical analysis of our dataset.

## Ethics Statement

The authors declare that there are no ethical issues with the data presented. The current work does not involve human subjects, animal experiments, or any data collected from social media platforms.

## CRediT authorship contribution statement

**Salwana Md. Jaafar:** Conceptualization, Methodology, Validation, Investigation, Data curation, Writing – original draft, Visualization. **Rahayu Sukmaria Sukri:** Conceptualization, Methodology, Validation, Writing – review & editing, Supervision, Project administration, Funding acquisition.

## Data Availability

Soil Physicochemical Properties Data in Bornean Tropical Heath Kerangas Forests Invaded by Acacia mangium (Original data) (Zenodo)
